# Risk for severe outcomes of COVID-19 and PIMS-TS in children with SARS-CoV-2 infection in Germany

**DOI:** 10.1007/s00431-022-04587-5

**Published:** 2022-08-13

**Authors:** Anna-Lisa Sorg, Markus Hufnagel, Maren Doenhardt, Natalie Diffloth, Horst Schroten, Rüdiger von Kries, Reinhard Berner, Jakob Armann

**Affiliations:** 1 Institute of Social Paediatrics and Adolescent Medicine, Division of Paediatric Epidemiology, Ludwig-Maximilians-University Munich, Munich, Germany; 2 University Children’s Hospital, Eberhard Karls University, Tuebingen, Germany; 3 Division of Pediatric Infectious Diseases and Rheumatology, Department of Pediatrics and Adolescent Medicine, University Medical Center, Medical Faculty, University of Freiburg, Freiburg, Germany; 4 Department of Pediatrics, University Hospital and Medical Faculty Carl Gustav Carus, Technische Universität Dresden, Dresden, Germany; 5 Pediatric Infectious Diseases, Department of Pediatrics, Medical Faculty Mannheim, Heidelberg University, Heidelberg, Germany

**Keywords:** COVID-19, Children, SARS-CoV-2, PIMS-TS, Risk, MIS-C

## Abstract

Although children and adolescents have a lower burden of SARS-CoV-2-associated disease compared to adults, assessing the risk for severe outcomes among SARS-CoV-2-infected children remains difficult due to a high rate of undetected cases. We combine data from three data sources — a national seroprevalence study (the SARS-CoV-2 KIDS study), the nationwide, state-based reporting system for PCR-confirmed SARS-CoV-2 infections in Germany, and a nationwide registry on children and adolescents hospitalized with either SARS-CoV-2 or pediatric inflammatory multisystem syndrome (PIMS-TS, also known as MIS-C) — in order to provide estimates on the risk of hospitalization for COVID-19-related treatment, intensive care admission, and death due to COVID-19 and PIMS-TS in children. The rate of hospitalization for COVID-19-related treatment among all SARS-CoV-2 seropositive children was 7.13 per 10,000, ICU admission 2.21 per 10,000, and case fatality was 0.09 per 10,000. In children without comorbidities, the corresponding rates for severe or fatal disease courses were substantially lower. The lowest risk for the need of COVID-19-specific treatment was observed in children aged 5–11 without comorbidities. In this group, the ICU admission rate was 0.37 per 10,000, and case fatality could not be calculated due to the absence of cases. The overall PIMS-TS rate was 2.47 per 10,000 SARS-CoV-2 infections, the majority being children without comorbidities.

*Conclusion*: Overall, the SARS-CoV-2-associated burden of a severe disease course or death in children and adolescents is low. This seems particularly the case for 5–11-year-old children without comorbidities. By contrast, PIMS-TS plays a major role in the overall disease burden among all pediatric age groups.

**Table:** 

**What is Known:** *• SARS-CoV-2-associated burden of disease in children is considered to be low, but accurate risk estimates accounting for clinically undiagnosed infections are lacking.* *• Asymptomatic SARS-CoV-2 infections are common in children.*	
**What is New:** *• We provide risk estimates for hospitalization for COVID-19-related treatment, ICU admission, death from COVID-19, and PIMS-TS for children with SARS-CoV-2 infections by pooling different data sources.* *• The risk for PIMS-TS exceeds the risk for severe COVID-19 in all age groups; the risk for severe COVID-19 is the lowest in 5–11 years old.*

**What is Known:**

*• SARS-CoV-2-associated burden of disease in children is considered to be low, but accurate risk estimates accounting for clinically undiagnosed infections are lacking.*

*• Asymptomatic SARS-CoV-2 infections are common in children.*

**What is New:**

*• We provide risk estimates for hospitalization for COVID-19-related treatment, ICU admission, death from COVID-19, and PIMS-TS for children with SARS-CoV-2 infections by pooling different data sources.*

*• The risk for PIMS-TS exceeds the risk for severe COVID-19 in all age groups; the risk for severe COVID-19 is the lowest in 5–11 years old.*

## Introduction

Since December 2019, severe acute respiratory syndrome coronavirus 2 (SARS-CoV-2) [1] has rapidly spread worldwide — by March 2020, the World Health Organization (WHO) had declared it a pandemic. As compared to adults [2], children and adolescents usually have mild disease courses, along with low disease-associated morbidity and mortality [3–5]. Nevertheless, their absolute risk of severe outcomes remains difficult to assess. Risk assessment requires accurate case numbers and knowledge of the actual population at risk — the number of SARS-CoV-2 infections. Due to a high rate of undetected cases among children [6], PCR-confirmed cases reported to state-based reporting systems significantly underestimate overall number of infections in this age group. Therefore, unless corrected accordingly, this can lead to a substantial overestimation of the risk of severe outcomes for children and adolescents. Additionally, a relevant number of patients with laboratory-confirmed SARS-CoV-2 infections during a hospital stay for different reasons may inflate the number of cases and thus the risk assessment even further [7].

Without reliable data on the actual infection-associated disease burden, a meaningful risk–benefit assessment for pandemic control and mitigation measures affecting this particular age group is impossible.

Here, we analyze information from a seroprevalence study in children — the SARS-CoV-2 KIDS study, recently published by Sorg et al. [8] — with publicly accessible data from the nationwide, state-based reporting system for PCR-confirmed SARS-CoV-2 infections in Germany on hospitalization and mortality [9], and data from a nationwide registry on hospitalized children with COVID-19 and pediatric inflammatory multisystem syndrome temporarily associated with SARS-CoV-2 (PIMS-TS, also known as MIS-C), the latter of which is sponsored by the German Society for Pediatric Infectious Diseases (DGPI) [10, 11]. By combining this data, we aimed to provide a risk estimates regarding hospitalization for COVID-19-related treatment, intensive care admission, and death due to COVID-19 and PIMS-TS for children of different age groups. The choice of age groups was made based upon vaccination eligibility in Germany.

## Material and methods

Our analysis is based upon information extracted from the results of three different surveys.

### SARS-CoV-2 KIDS study

The proportion of children with positive titers against SARS-CoV-2 immunoglobulin G (IgG) was recently published for the SARS-CoV-2 KIDS study [8]. This is a hospital-based, multi-center, longitudinal study in children aged ≤ 17 years. From June 2020 to May 2021, in 14 pediatric hospitals across Germany, 10,358 participants were recruited during their inpatient or outpatient stays (monthly average recruiting rate: 863 (± SD 220) participants). Blood samples taken for routine clinical procedures were additionally tested for the IgG-specific S1 domain of SARS-CoV-2 spike protein by means of an enzyme-linked immunosorbent assay (ELISA – Euroimmun Medizinische Diagnostika AG, Lübeck, Germany). Children with corrected gestational age less than 37 completed weeks, severe congenital or acquired immune deficiencies, immunosuppression due to chemotherapy or stem cell transplantation, treatment due to life-threatening emergencies, and children already vaccinated against SARSCoV-2 were excluded from participation.

The SARS-CoV-2 KIDS study authors reported a seroprevalence of SARS-CoV-2 IgG antibodies of 10.8% (95% CI 8.7, 12.9) for March 2021, and observed no major change through May 2021 [8]. Additionally, by the end of the observation period, they detected no differences in seroprevalence among the different age groups.

The German Federal Statistical Office provides updated data on the total population of children in Germany and the breakdown of age groups [12]. The seroprevalence estimates included in the SARS-CoV-2 KIDS study publication were extrapolated for the total population.

### DGPI registries

On March 2020, near the beginning of the pandemic, a national, prospective registry for children and adolescents hospitalized with a SARS-CoV-2 infection in Germany was established. On May 28, 2020, it was expanded to also capture PIMS-TS cases. All German pediatric hospitals were invited to participate. Included for analysis were any hospitalized patients up to 17 years old who had laboratory-confirmed SARS-CoV-2 infections, (reported to the registry from March 1, 2020, to May 31, 2021), as well as those who fulfilled the WHO case definition for PIMS-TS [13] (reported to the registry from May 28, 2020, to May 31, 2021).

Via a link on the DGPI website (https://dgpi.de/covid-19-survey-der-dgpi/), access was provided to an electronic case report form on a REDCap (Research Electronic Data Capture) platform hosted at Technische Universität Dresden. Data collected included demographic characteristics, symptoms and clinical signs, treatments, disease course during hospitalization, and outcome at hospital discharge. The data of the cases cover the time from the positive test result until death or discharge from hospital. Hospitalized cases comprised children in hospital for COVID-19 treatment (hospitalization for COVID-19-related treatment) and SARS-CoV-2-positive children hospitalized for other reasons. COVID-19-releated treatment was defined by the reporting physician of each case and includes any therapeutic intervention in the hospital aimed towards SARS-CoV-2 — medications (in the majority of cases symptomatic treatment as nasal decongestants, antipyretics, or intravenous fluids) as well as respiratory support. Children hospitalized for other reasons were those cases, where no treatment in relation to the SARS-CoV-2 infection was reported. We defined severe outcomes of SARS-CoV-2 infection as hospitalization for COVID-19-related treatment, admission to an intensive care unit, death due to COVID-19 defined by the reporting physician, or PIMS-TS.

#### Nationwide, state-based reporting notification system for PCR-confirmed SARS-CoV-2 infections in Germany

The third type of survey included data from the nationwide, state-based reporting system of laboratory-confirmed SARS-CoV-2 infections in Germany [9]. In Germany, laboratory confirmation requires detection of SARS-CoV-2 nucleic acid by PCR or by culture isolation of the pathogen (according to the national case definition [14]). There is a legal obligation to report cases tested positive for SARS-coV-2 to the local public health authorities (PHA), who then forward the information via the respective state PHA to the Robert Koch-Institute (RKI – Germany’s national public health institute), in Berlin. Reporting includes information on hospitalization status and mortality. Data on hospitalization and death by age group up to the age of 18 were kindly provided by the RKI. To estimate potential underreporting in the DGPI registry (voluntary reporting), we compared the PCR-confirmed case numbers from the nationwide, state-based reporting system for PCR-confirmed SARS-CoV-2 infections in Germany (compulsary) with those from the DGPI registry regarding hospitalized children with a diagnosis of SARS-CoV-2 infection. Unfortunately, the state-based reporting system does not allow to distinguish between cases treated in hospital because of or with a SARS-CoV-2 infection. Therefore, correction for underreporting for severe outcomes for COVID-19 in hospitalized children had to be based on correction for reporting of all SAR-CoV-2 infections identified in any hospitalized children.

### Outcomes of interest

To assess the morbidity and mortality risk to children infected with SARS-CoV-2, we assessed five outcome measurements:

1. Hospitalization for COVID-19-related treatment: the number of hospitalized patients requiring any form of therapeutic intervention for COVID-19 (as defined by the physician who reported the case) in the DGPI registry
2. COVID-19 requiring admission to intensive care unit (ICU) in the DGPI registry
3. Death associated with COVID-19: the number of case fatalities due to COVID-19 was based upon the total reported in the state-based reporting system for PCR-confirmed SARS-CoV-2 infections in Germany
4. Hospitalization associated with PIMS-TS: patients hospitalized due to PIMS-TS reported in DGPI registry
5. ICU stay associated with PIMS-TS: patients admitted to ICU due to PIMS-TS

### Statistical analysis

To calculate the risk for the different outcome measurements, we estimated the number of SARS-CoV-2-infected children by using the number of children in the respective age group as reported by the Federal Statistical Office and the SARS-CoV-2 KIDS Study data [8]. To account for sampling imprecision, the upper and lower limits of the reported 95% confidence intervals served as a denominator for the risk estimate limits regarding the SARS-CoV-2 outcome measurements.

In order to account for underreporting in the DGPI registry, we multiplied the numbers of COVID-19 patients requiring therapeutic interventions and ICU admissions due to COVID-19 by the rate of underreporting. Underreporting was calculated by dividing the age strata specific number of hospital cases in the state-based reporting system (n_S_) by the respective numbers reported to the DGPI registry (n_D_). From March 2020 to May 2021, 1620 children were reported in the DGPI registry and 5780 the state-based reporting system. This allowed us to calculate an underreporting rate of 2.49 (2421/972) for the < 5 years old, 7.30 (1278/175) for the 5 to 11 years old, and 4.40 (2081/473) for the 12 to 17 years old.

We did not adjust the overall number of PIMS-TS cases, because these cases are not reported in the state-based reporting system. To date, there has been no other source for data on PIMS-TS in Germany.

The number of children living in Germany without comorbidities is unknown. Information on medical history was available for the cases reported via the DGPI registry. To estimate the outcome risks for the children for with no medical history, we reduced the population size by the proportion of children with comorbidities, as reported in the DGPI registry (28.8%). On this basis, we postulated that 71.2% of the seropositive population would have no relevant comorbidities.

The age range groupings were selected according to COVID-19 vaccination recommendations for children in Germany: children “under 5 years of age,” children “5 to 11 years of age,” and children “12 to 17 years of age.” At first, vaccination was only recommended for children with pre-existing conditions in Germany, which was replaced by general vaccination recommendations (June 2021: children 12–17 years old with pre-existing diseases; August 2021: all children 12–17 years; January 2022: children 5–11 years old with pre-existing diseases; May 2022: all children; up to know: no available licensed vaccine for children younger < 5 years in Germany) [15]. In the observation period of this analysis, there was no recommendation to vaccinate children in Germany. Chi-square tests were used to examine the differences between the age groups and between the groups with and without comorbidities.

Our analysis was performed using SAS Version 9.4 (SAS Institute, Cary, NC, USA). The significance level was set at 5%.

## Results

As of December 31, 2020, approximately 13.7 million people in Germany were < 18 years old [12]. With a seroprevalence of 10.8% (95 CI 8.7, 12.9) on May 2021 (as estimated by the SARS-CoV-2 KIDS study) and without substantial differences according to age groups, 1,484,346 (1,195,723; 1,772,969) children are likely to have been in contact with the virus. As such, they form a population at-risk for hospitalization for COVID-19 related treatment, ICU admission, and/or death due to COVID-19 or PIMS-TS.

**Fig. 1:**
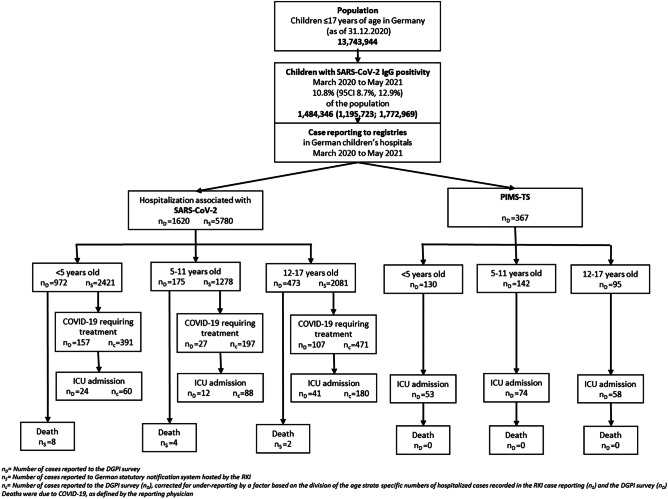
Flow chart of pediatric cases with COVID-19 and PIMS-TS hospitalized reported in the different registries from March 2020 to May 2021 — number of reported hospitalizations, hospitalizations with COVID-19-related treatment, and ICU admission or death

The flowchart displays the reported number of the respective outcomes reported by the DGPI survey (n_D_), the state-based reporting system (n_S_), and the numbers adjusted for underreporting (n_c_), where appropriate. The corrected numbers for hospitalization for SARS-CoV-2-related treatment were 197 in the 5–11-year-old age group and 471 in the 12–17-year-old group. In both age groups, the absolute numbers were markedly reduced when limited to cases requiring ICU admission (Fig. 1).

### Risks associated with SARS-CoV-2 infection

As of May 2021, 291 children with hospitalization for COVID-19-related treatment had been reported in the DGPI survey. Taking possible under-reporting into account, the rate of hospitalized children with COVID-19 requiring treatment was 7.13 (5.97; 8.86) per 10,000 children and 2.21 (1.85; 2.74) for COVID-19 cases admitted to the ICU (Table 1).

**Table 1:** Risk associated with SARS-CoV-2-infection in children: severe COVID-19 and PIMS-TS

**Age category** (in years)	Population at risk*	**SARS-CoV-2 IgG positivity** 10.8% (95 CI 8.7, 12.9) of the population ≤ 17 years	**Hospitalization for COVID-19-related treatment**		**COVID-19-related ICU admission**		Death due to COVID-19^**^		**PIMS-TS**		**PIMS-TS ICU admission**	
	*n*	*n* (95 CI)	*n*_*c*_	Risk per 10,000	*n*_*c*_	Risk per 10,000	*n*_*S*_	Risk per 10,000	*n*_*D*_	Risk per 10,000	*n*_*D*_	Risk per 10,000
**Total**	**13,743,944**	**1,484,346 (1,195,723; 1,772,969)**	**1059**	**7.13 (5.97; 8.86)**	**328**	**2.21 (1.85; 2.74)**	**14**	**0.09 (0.08; 0.12)**	**367**	**2.47 (2.07; 3.07)**	**185**	**1.25 (1.04; 1.55)**
< 5	3,969,138	428,667 (345,315; 512,019)	391	9.12 (7.64; 11.32)	60	1.40 (1.17; 1.74)	8	0.19 (0.16; 0.23)	130	3.03 (2.54; 3.76)	53	1.24 (1.04; 1.53)
5–11	5,267,742	568,916 (458,294; 679,539)	197	3.46 (2.90; 4.30)	88	1.55 (1.29; 1.92)	4	0.07 (0.06; 0.09)	142	2.50 (2.09; 3.10)	74	1.30 (1.09; 1.61)
12–17	4,507,064	486,763 (392,115; 581,411)	471	9.68 (8.10; 12.01)	180	3.70 (3.10; 4.59)	2	0.04 (0.03; 0.05)	95	1.95 (1.63; 2.42)	58	1.19 (1.00; 1.48)

*n*_*D*_ number of cases reported to the DGPI survey, *n*_*S*_ number of cases reported to the nationwide, state-based reporting notification system for OCR-confirmed SARS-CoV-2 infections in Germany hosted by the RKI, *n*_*c*_ number of cases reported to the DGPI survey (n_D_), corrected for under-reporting by a factor based on the division of the age strata specific numbers of hospitalized cases recorded in the RKI case reporting (n_S_) and the DGPI survey (n_D_)

**Death due to COVID-19 defined by the reporting physician

The rate of hospitalization for COVID-19 treatment was lowest for the age group 5–11 years (*p* < 0.0001). Regarding intensive care treatment, the rate was highest in children aged 12 to 17 years (*p* < 0.0001) compared to the other age groups.

Our analysis found a case fatality of 0.09 per 10,000 children through May 2021 in Germany. This was based upon a total number of 14 pediatric fatalities due to COVID-19. The DGPI registry captured almost all of these fatalities, with 13 reported cases as compared to the 14 recorded in the state-based reporting system. In 5/13 (38%) of these cases, the patients had been in palliative care due to an underlying disease prior to their SARS-CoV-2 infection.

Limiting the analysis to children without comorbidities decreased the estimated risk of treatment-requiring COVID-19 hospitalizations to 5.19 per 10,000 and the risk of ICU admission due to COVID-19 to 0.90 per 10,000 children (Table 2). Comorbidity is significantly associated with COVID-19-related treatment and ICU admission due to COVID-19 (both *p*-values < 0.0001). Among children without comorbidities, case fatality was 0.03 per 10,000, with no deaths reported in children ≥ 5 years of age.

**Table 2:** Risk associated with SARS-CoV-2-infection in children without comorbidities: severe COVID-19 and PIMS-TS

**Age category** (in years)	Population at risk*	**SARS-CoV-2 IgG positivity** 10.8% (95 CI 8.7, 12.9) of the population ≤ 17 years	**Hospitalization for COVID-19-related treatment**		**COVID-19-related ICU admission**		Death due to COVID-19^**^		**PIMS-TS**		**PIMS-TS ICU admission**	
	*n*	*n* (95 CI)	*n*_*c*_	Risk per 10,000*	*n*_*c*_	Risk per 10,000*	*n*_*S*_	Risk per 10,000*	*n*_*D*_	Risk per 10,000*	*n*_*D*_	Risk per 10,000*
**Total**	**9,780,335**	**1,056,276 (850,889; 1,261,663)**	**548**	**5.19 (4.34; 6.44)**	**95**	**0.90 (0.75; 1.12)**	**3**	**0.03 (0.02; 0.04)**	**311**	**2.94 (2.47; 3.66)**	**163**	**1.54 (1.29; 1.92)**
< 5	2,824,480	305,044 (245,730; 364,358)	274	8.98 (7.52; 11.15)	27	0.89 (0.74; 1.10)	3	0.10 (0.08; 0.12)	117	3.84 (3.21; 4.76)	51	1.67 (1.40; 2.08)
5–11	3,748,580	404,847 (326,126; 483,567)	80	1.98 (1.65; 2.45)	15	0.37 (0.31; 0.46)	0	-	121	2.99 (2.5;0 3.71)	65	1.61 (1.34; 1.99)
12–17	3,207,274	346,386 (279,033; 413,738)	194	5.60 (4.69; 6.95)	53	1.53 (1.28; 1.90)	0	-	73	2.11 (1.76; 2.62)	47	1.36 (1.14; 1.68)

*n*_*D*_ number of cases reported to the DGPI survey, *n*_*S*_ number of cases reported to the nationwide, state-based reporting notification system for OCR-confirmed SARS-CoV-2 infections in Germany hosted by the RKI, *nc* number of cases reported to the DGPI survey (n_D_), corrected for under-reporting by a factor based on the division of the age strata specific numbers of hospitalized cases recorded in the RKI case reporting (n_S_) and the DGPI survey (n_D_)

*Children ≤ 17 years living in Germany reduced by a proportion of 28.8% (expected maximum number of children with pre-existing conditions, derived from the DGPI data, where 462 out of 1602 children had pre-existing conditions); **Death due to COVID-19 defined by the reporting physician

For the overall study cohort, the cumulative risk for developing PIMS-TS was 2.47 per 10,000 and 1.25 per 10,000 for ICU admission due to PIMS-TS (Table 1). Significantly fewer cases of PIMS-TS were reported in 12–17 years old compared to the other age groups (*p* = 0.005), but there were no age differences in ICU admissions due to PIMS-TS (*p* = 0.88). Confining the analysis to children without comorbidities slightly increased the overall risk to 2.94 per 10,000 for PIMS-TS and to 1.54 per 10,000 for ICU admission due to PIMS-TS (Table 2).

## Discussion

By combining the results of a nationwide seroprevalence study [8] together with data from clinical registries and the nationwide, state-based reporting system for PCR-confirmed SARS-CoV-2 infections in Germany, we were able to provide risk estimates of the SARS-CoV-2-associated disease burden, both for the acute infection phase and for PIMS-TS, a hyperinflammatory syndrome that typically usually occurs 4–6 weeks after a mild or asymptomatic SARS-CoV-2 infection.

Overall, the extremely low risk for severe disease course — as indicated by the need for in hospital therapeutic intervention, admission to ICU or death during the acute infection phase — is consistent with data previously reported from other countries [16, 17]. Consistent with data provided by the CDC [18], children in the 5–11 age group seem to have a lower risk than children < 5 years and adolescents 12–17 years of age.

Unfortunately, we have no information on the variants of the reported SARS-CoV-2 cases. The contribution of different variants can only be roughly estimated from the varying pattern of circulating variants in German over time. We assume that the majority of cases are attributable to the alpha (B.1.1.7) variant, since the delta (B.1.617.2) variant was first detected in Germany at the end of April 2021 and only increased very strongly from the end of May 2021 [19].

With emergence of the Omicron variant, severe clinical outcomes became significantly less frequent [20]. The absolute risk for children and adolescents infected with the currently circulating variants will therefore be even lower than reported in this analysis.

Interestingly, children without comorbidities account for more than half of all COVID-19 cases requiring therapeutic interventions (548 of 1059 cases) and one-third of the cases requiring ICU admission (95 of 328 cases). A considerable part is attributable to children with comorbidities, even though they make up only a small portion of the pediatric population. Primarily, healthy children ages 5–11 have the lowest risk with a 0.37/10,000 rate of ICU admission. Due to an absence of cases, their case fatality rate cannot be calculated.

A study from the USA using a similar methodology approach found an overall risk for PIMS-TS of 316 (95% CI, 278–357) per 1,000,000 SARS-CoV-2 infections, but with variabilities between the states [21]. With our data, we found a similar overall risk for PIMS-TS of about 250 per 1,000,000. With about 50% of these cases requiring ICU admission, PIMS-TS plays a relevant role in the overall SARS-CoV-2-associated disease burden in the pediatric age group. It leads to about a quarter of all treatment-requiring hospitalizations and almost 40% of all ICU admission. In children without comorbidities, the role of PIMS-TS is even more pronounced and accounts for 38% of all treatment-requiring hospitalizations and 65% of ICU admissions. The US study also showed age differences considering PIMS-TS with likewise lowest incidence rates among the adolescents [21]. The latter, however, might be biased since our and the US study were based on reports mostly from pediatric hospitals and some adolescent cases might be treated in adult facilities.

Fortunately, there are effective treatment options for PIMS-TS. Only small numbers of patients have suffered from sequalae and follow-up data emerging from the UK and the USA has been reassuring [22–24]. In addition, both national [11] and international [25] PIMS-TS registries detect a lower rate of PIMS-TS cases, as the delta-variant and then especially when the omicron has predominated. In fact, when analyzing data from the DGPI PIMS registry, we could see a 70–90% risk reduction with omicron compared to alpha [26]. This data is important to consider, especially given the role that PIMS-TS plays in the overall SARS-CoV-2-associated disease burden among children and adolescents.

### Strengths and limitations

The strength of our analysis lies in its provision of estimates for evaluating the risk of severe manifestations related to SARS-CoV-2 infections in children based on SARV-CoV-2 seropositivity instead of PCR-confirmed case numbers of state-based reporting system. Although seropositivity is likely to be the best available surrogate marker for the time course of SARS-CoV-2 infections, there are some limitations: potential sampling variability, waning of antibodies, and unclear sensitivity. We addressed sampling variability by providing the 95% CI. Due to waning of antibodies, it could be that by using seropositivity, we are also underestimating the number of infections. This would result in the risk estimators presented being overestimated. However, so far, available data on waning in children suggest that titers do not decline even months after infection [27, 28].

The size of the population at risk in absence of comorbidities may be somewhat overestimated because infected children with risk factors are more likely to be hospitalized. Unfortunately, we had no better estimator for prevalence of comorbidities in the general population.

Reporting to the DGPI register is voluntary, while reporting laboratory-confirmed SARS-CoV-2 infections to the state-based reporting system is compulsory. Thus, the latter may be assumed to be more comprehensive. Because case definitions for reporting hospitalized cases in the DGPI survey and the state-based reporting system were identical, this provides a reasonable basis for correcting the underreporting of the DGPI survey. Unfortunately, due anonymous reporting, we could not link the two data sources, precluding capture-recapture calculation. Although we considered corresponding and stratified data for both data sources for the entire age group, the correction factor may not be equally valid for all degrees of severity of COVID-19. While fine for the number of any hospitalization, some overestimations appear possible regarding hospitalization for COVID-19-related treatment and likely for cases with ICU admission, in case the probability reporting to DGPI increases by severity of disease. The number of deaths reported in both systems, indeed, was almost equal.

Regarding the risk for PIMS-TS, which could not be corrected since the state-based reporting system does not record PIMS-TS, we cannot exclude under-reporting. But we found a risk similar to that reported in the literature.

Our definition of relevant cases as cases which require in hospital COVID-19-related treatment was chosen with respect to the wide specter of — mostly unspecific — clinical symptoms in children. Hospital COVID-19-related treatment was defined by the reporting physician who definitely can identify which therapeutic interventions are for the SARS-CoV-2 infection or not.

We chose to report the corrected risk estimates, which reflect estimates for the maximal possible risk for severe outcomes of COVID-19 infections in children. This is an important figure for decision-making, indicating, e.g., the maximum possible reduction of severe COVID-19 morbidity with a 100% effective vaccine and complete uptake.

## Conclusion

Our analysis of the data shows that the SARS-CoV-2-associated burden of severe disease or death in children and adolescents is low. This is particularly true for COVID-19-related symptoms in 5–11-year-old children without comorbidities. PIMS-TS appears to play a crucial role in overall SARS-CoV-2-associated disease burden and exceeds the risks of the acute severe COVID-19 children and adolescents without comorbidities.

## Data Availability

All data relevant to the study are included in the article.

## Abbreviations

COVID-19: Coronavirus disease 2019
DGKJ: German Society of Pediatrics and Adolescents Medicine
DGPI: German Society for Pediatric Infectious Diseases
ELISA: Enzyme-linked immunosorbent assay (Euroimmun Medizinische Diagnostika AG, Lübeck, Germany)
ICU: Intensive care unit
IgG: Immunoglobulin G
PCR: Polymerase chain reaction
PHA: Public health authorities
PIMS-TS: Pediatric inflammatory multisystem syndrome associated with COVID-19
REDCap: Research Electronic Data Capture
RKI: Robert Koch-Institute
SARS-CoV-2: Severe acute respiratory syndrome coronavirus type 2
VLKKD: Association of Senior Pediatricians and Pediatric Surgeons in Germany
WHO: World Health Organization

## Code availability

The code used to generate the results is available on request from the corresponding author.
